# Malignant paraganglioma of the kidney: a rare surgical case with 3-year disease-free survival

**DOI:** 10.1093/jscr/rjaf494

**Published:** 2025-07-11

**Authors:** Fu-Xiang Lin, Pengpeng Zhao, Le Xie, Zhan-Ping Xu

**Affiliations:** The Eighth Clinical Medical College of Guangzhou University of Chinese Medicine, No. 6 Qinren Road, Foshan 528000, Guangdong, People’s Republic of China; Department of Urology, Foshan Hospital of Traditional Chinese Medicine, No. 6 Qinren Road, Foshan 528000, Guangdong, People’s Republic of China; The Eighth Clinical Medical College of Guangzhou University of Chinese Medicine, No. 6 Qinren Road, Foshan 528000, Guangdong, People’s Republic of China; Department of Urology, Foshan Hospital of Traditional Chinese Medicine, No. 6 Qinren Road, Foshan 528000, Guangdong, People’s Republic of China; The Eighth Clinical Medical College of Guangzhou University of Chinese Medicine, No. 6 Qinren Road, Foshan 528000, Guangdong, People’s Republic of China; Department of Pathology, Foshan Hospital of Traditional Chinese Medicine, No. 6 Qinren Road, Foshan 528000, Guangdong, People’s Republic of China; The Eighth Clinical Medical College of Guangzhou University of Chinese Medicine, No. 6 Qinren Road, Foshan 528000, Guangdong, People’s Republic of China; Department of Urology, Foshan Hospital of Traditional Chinese Medicine, No. 6 Qinren Road, Foshan 528000, Guangdong, People’s Republic of China

**Keywords:** malignant paraganglioma, renal tumor, radical nephrectomy, adrenalectomy, case report

## Abstract

A 36-year-old woman presented with abdominal pain and a left renal mass on imaging. Laboratory findings showed anemia (Hb 88 g/l), leukocytosis (12.31 × 10^9^/l), and pyuria. Computed tomography (CT) revealed a 14 cm left renal cystic-solid mass suspicious for malignancy. Radical nephrectomy was performed, retrieving a 2600 g tumor. Histopathology demonstrated malignant paraganglioma with expansive growth confined to renal parenchyma. No adjuvant therapy was administered. At 3-year follow-up, surveillance positron emission tomography-computed tomography (PET-CT) confirmed no recurrence or metastasis. Renal paragangliomas represent <1% of such tumors, with malignant variants posing diagnostic and therapeutic challenges. This case underscores surgical resection as definitive management for localized disease and suggests favorable outcomes are achievable without adjuvant treatment in select malignant cases. Long-term surveillance remains critical.

## Introduction

Paragangliomas are neuroendocrine tumors arising from autonomic nervous system ganglia, with >85% occurring in the adrenal medulla. Primary renal involvement is exceptionally rare, accounting for <1% of cases [1]. Malignant transformation occurs in <10% overall, but remains poorly characterized at renal sites due to scant literature [2]. We detail a surgically managed malignant renal paraganglioma with sustained remission at 3 years, examining implications for management strategies.

## Case report

**Figure 1:**
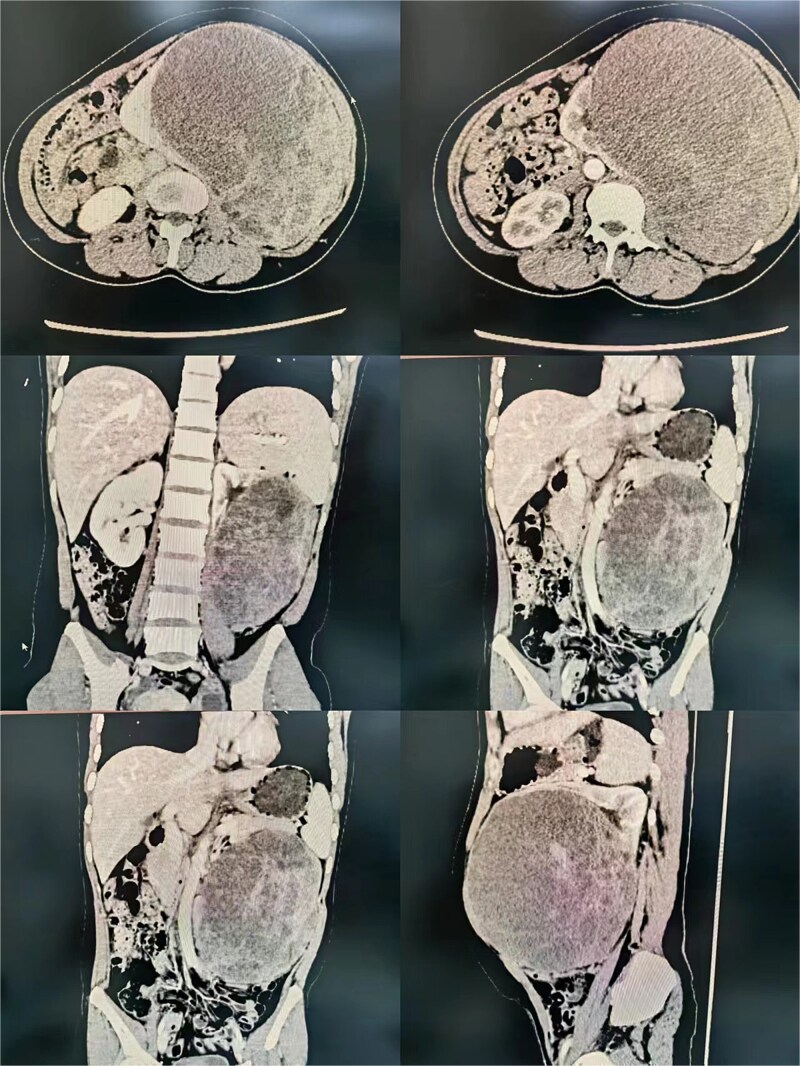
Radiological imaging of the left renal mass.

A 36-year-old female with no comorbidities presented with progressive abdominal pain for 1 month. Initial imaging suggested pancreatic origin, but contrast-enhanced CT localized a 14 × 13 × 12 cm heterogeneous left renal mass concerning for malignancy (Fig. 1). Laboratory workup revealed anemia (Hb 88 g/l), leukocytosis (12.31 × 10^9^/l), and sterile pyuria (WBC 814.1/μl). No hormonal hypersecretion was documented.

## Surgical procedure

**Figure 2:**
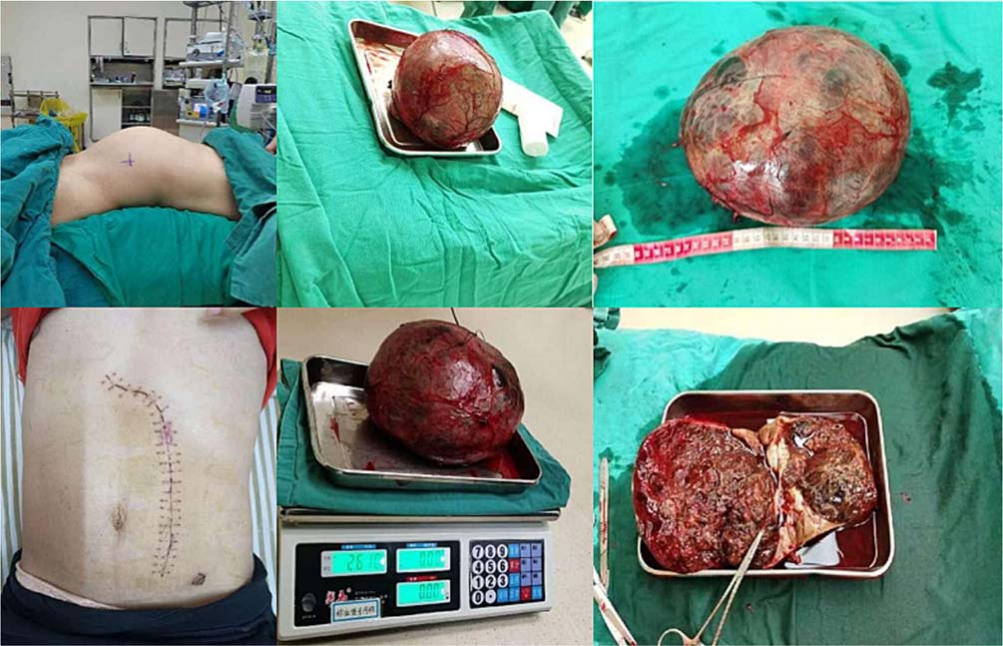
Gross image of the resected left renal mass.

Following right lateral decubitus positioning, a transperitoneal approach via left paramedian incision exposed a retroperitoneal mass displacing bowel superiority. Intraoperative findings confirmed renal parenchymal origin without adjacent organ

involvement. The renal artery and vein were sequentially ligated using surgical clips and silk sutures. Complete resection required en bloc nephrectomy with ureteral division. Operative time was 180 minutes with 500 ml blood loss necessitating transfusion (Fig. 2).

## Pathologic findings

**Figure 3:**
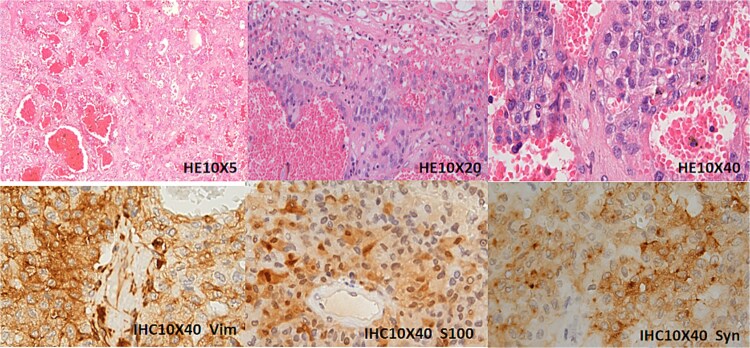
Immunohistochemical analysis of the left renal mass. The tumor is situated within the renal parenchyma, compressing and displacing adjacent renal tubules with associated atrophy. Histologically, it demonstrates nested and papillary growth patterns within a richly vascularized stroma. High-power examination reveals tumor cells with vacuolated or eosinophilic cytoplasm, prominent small nucleoli, and rare mitotic figures. Immunohistochemical staining shows diffuse cytoplasmic positivity for Vimentin (Vim), S100, and Synaptophysin (Syn).

Gross examination revealed a 2600 g encapsulated mass replacing 80% of renal parenchyma (Fig. 2). Histopathology demonstrated nested neoplastic cells with eosinophilic cytoplasm, nuclear atypia, and vascular invasion. Immunohistochemistry confirmed synaptophysin (+), chromogranin A (+), and Ki-67 index 15% (Fig. 3). These features supported malignant paraganglioma (World Health Organization Grade 1) with negative resection margins.

## Discussion

This report highlights three critical aspects of renal paraganglioma management.

### Diagnostic considerations

Renal paragangliomas frequently mimic renal cell carcinoma or retroperitoneal sarcomas radiographically. In our case, initial misattribution to pancreatic origin underscores diagnostic pitfalls. Preoperative biopsy is discouraged due to bleeding risk and sampling limitations [3]. Elevated leukocyte count observed herein may reflect tumor necrosis, previously unreported in renal cases.

### Surgical strategy

Radical nephrectomy remains the cornerstone treatment [4]. The transperitoneal approach facilitated safe dissection despite tumor bulk, avoiding intraoperative hemodynamic instability seen in functional pheochromocytomas. Unlike adrenal paragangliomas requiring adrenal vein-first ligation, renal venous control followed arterial occlusion without complications.

### Long-term outcomes

Our patient’s 3-year disease-free status contrasts with reported 40%–50% metastatic rates in malignant cases [5]. This discrepancy may reflect complete excision before vascular spread. The 15% Ki-67 index fell below the 20% cutoff predicting aggressive behavior in extra-adrenal paragangliomas [5], supporting surveillance-only post-resection. Serial PET-CT remains essential given late recurrence risks.

## Conclusion

Malignant renal paraganglioma, though rare, should be considered in large hypervascular renal masses. Open nephrectomy provides definitive therapy with potential for extended recurrence-free survival even in malignant variants. Avoiding adjuvant treatment in cases with favorable histological features warrants a prospective study.
